# Size‐selective fishing and the potential for fisheries‐induced evolution in lake whitefish

**DOI:** 10.1111/eva.12635

**Published:** 2018-04-25

**Authors:** Yolanda E. Morbey, Marin Mema

**Affiliations:** ^1^ Department of Biology Western University London Ontario Canada

**Keywords:** fisheries‐induced evolution, freshwater fisheries, growth rate, Lake Huron, life history evolution, maturation reaction norm

## Abstract

The long‐term evolutionary effects of fishing on maturation schedules can depend on gear type, the shape of the gear type's size‐selectivity function, and the size and age structure of a population. Our goal was to better understand how environmentally induced differences in somatic growth influence the evolutionary effects of size‐selective fisheries, using lake whitefish (*Coregonus clupeaformis*) in Lake Huron as a case study. Using a state‐dependent optimization model of energy allocation parameterized for lake whitefish, we show that fishing with gill nets (bell‐shaped selectivity) and trap nets (sigmoid‐shaped selectivity) can be potent agents of selection on size thresholds for maturity. Compared to trap nets and large mesh (114 mm) gill nets, small mesh (89 mm) gill nets are better able to buffer populations from fishing‐induced evolution by safeguarding large, fecund fish, but only when overall fishing mortality is low and growth rates sufficiently fast such that fish can outgrow vulnerable size classes. Regardless of gear type, and all else being equal, high fishing mortality in combination with low growth rates is expected to intensify the long‐term evolutionary effects of fishing.

## 1 INTRODUCTION

A central tenet of life history theory is that optimal age and size at maturity reflect a balance between the benefits of growing larger and achieving higher reproductive potential and the costs of dying before realizing that reproductive potential (Roff, 1984; Stearns, 1992). Thus, fishing mortality, which can target a large range of ages and sizes, is expected to be an important selection factor on life histories in exploited fish populations, leading to evolutionary changes in growth rate, size and age thresholds for maturation, and fecundity (Dunlop, Enberg, Jørgensen, & Heino, 2009; Heino, Pauli, & Dieckmann, 2015; Jørgensen et al., 2007; Kuparinen & Merilä, 2007). Indeed, evidence has emerged that fishing mortality of stocks, both marine and freshwater, can be sufficiently high for evolutionary changes in life history traits to occur at trackable, ecological timescales (Edeline et al., 2007; Heino et al., 2015; Jørgensen et al., 2007; Nusslé, Bornand, & Wedekind, 2009; Olsen et al., 2004). Further, the direction and intensity of fisheries‐induced selection are expected to depend on which sizes are targeted, either through size or gear restrictions (Dunlop, Heino, & Dieckmann, 2009; Hutchings, 2009; Jørgensen, Ernande, & Fiksen, 2009; Wang & Höök, 2009). For example, Jørgensen et al. (2009) demonstrated that trawl nets, which have sigmoid‐shaped selectivity curves, consistently favor early maturation in Atlantic cod (*Gadus morhua*). In contrast, gill nets, which have bell‐shaped size selectivity curves, can select for fish that grow through vulnerable size classes before maturing. This outcome has the potential to reduce fisheries‐induced selection for early maturation. For any specific fishery stock, however, this outcome likely will depend on somatic growth rate and the potential for fish to grow through vulnerable stages quickly. Our goal was to better understand how the growth regime influences the evolutionary effects of size‐selective fisheries in lake whitefish (*Coregonus clupeaformis*).

Lake whitefish populations have long experienced fishing mortality and variable growth regimes. In the Laurentian Great Lakes, lake whitefish support subsistence and commercial fisheries, and historically faced high exploitation rates in commercial gill net and trap net fisheries (Brenden, Brown, Ebener, Reid, & Newcomb, 2013; Ebener, Brenden, & Jones, 2010; Ebener et al., 2008). Ebener et al. (2008) provide an historical account of lake whitefish fisheries in the Great Lakes (also see Eberts et al., 2017). High exploitation rate in combination with other factors led to the collapse of lake whitefish fisheries throughout the Great Lakes from 1955 to 1970. Subsequent management actions then led to the recovery of lake whitefish populations and the reestablishment of large commercial fisheries. However, following the invasion of zebra and quagga mussels *(Dreissena polymorpha* and *D. bugensis*) and loss of *Diporeia* spp. amphipods beginning in the 1990s, lake whitefish underwent significant changes in diet and associated declines in growth and recruitment in affected regions (Gobin, Lester, Cottrill, Fox, & Dunlop, 2015; Pothoven & Madenjian, 2008; Pothoven, Nalepa, Schneeberger, & Brandt, 2001; Pothoven et al., 2006; Rennie, Sprules, & Johnson, 2009; Rennie & Verdon, 2008). Particularly in some regions of Lake Huron, food web changes in combination with high sea lamprey (*Petromyzon marinus*) mortality have contributed to very low fishing effort and yields in recent years (Caroffino & Lenart, 2017).

Prevailing growth and mortality regimes drive patterns of life history variation among lake whitefish populations. Across their range in North America, lake whitefish populations show extensive variation in growth, mortality, and age and size at maturity (Beauchamp, Collins, & Henderson, 2004). For example, maximum total length ranges from 470 to 818 mm and maximum weight ranges from 1,140 to 11,460 g among 419 populations (Rennie & Verdon, 2008). Among populations, faster prereproductive growth correlates with earlier age at maturity, which is consistent with life history theory (Charnov, 1993). Within the Laurentian Great Lakes, large‐scale spatial variation in size thresholds for maturation among basins and lakes (e.g., smaller size thresholds in Lake Superior) has been hypothesized to result from local adaptation to large‐scale spatial variation in prevailing growth and mortality regimes (Wang, Höök, Ebener, Mohr, & Schneeberger, 2008).

The idea that environmental variation in growth potential interacts with size‐selective fisheries to affect the evolution of maturation traits is not new, yet this interaction has not been the focus of modeling studies. Certainly, all models of fisheries‐induced evolution of maturation traits include somatic growth rate as an input variable. Growth has been formulated in different ways in these models, but typically is not manipulated to generate predictions. In Atlantic cod, several modeling studies have considered the evolutionary and demographic consequences of size‐selective fisheries and different gear types under a specified growth regime (Hutchings, 2009; Jørgensen et al., 2009; Zimmerman & Jørgensen, 2017). In eco‐genetic models of fisheries‐induced evolution of maturation traits in lake whitefish, somatic growth rate is assumed to be negatively density‐dependent according to a specified functional form. This allows for feedbacks between ecological and evolutionary processes in response to alternative forms and intensities of size‐selective fishing (Dunlop, Eikeset, & Stenseth, 2015; Dunlop, Heino et al., 2009; Wang & Höök, 2009). Eco‐genetic modeling also has been used to explore how different intensities of density‐dependent growth affect population dynamics and stock productivity (Gobin, Lester, Fox, & Dunlop, 2016). Intrinsic growth rate also has been treated as a decision variable in models of size‐selective fisheries (Dunlop, Heino et al., 2009; Eikeset et al., 2016).

To better understand how environmental variation in growth might influence the evolutionary response to size‐selective fisheries, we focussed on two lake whitefish stocks in Lake Huron with long‐term assessment data. Our first objective was to characterize and compare the stock‐specific growth regimes and maturation schedules of lake whitefish. We reasoned that if stocks differ in their growth and maturation traits, they might also differ in their evolutionary response to size‐selective fishing. Our second objective was to model the influence of fishing mortality rate, different gear types, and growth regimes on optimal maturation schedules of lake whitefish. This was done using a state‐dependent optimization model of energy allocation that was informed by observational data. In our model, we vary the type of fishing gear, fishing mortality rate, and somatic growth rate.

## 2 METHODS

### 2.1 Lake whitefish data

Our first objective was to characterize cohort‐specific maturation traits, prematuration growth rates, and their relationship in Lake Huron lake whitefish. We used long‐term monitoring data from the Ontario Ministry of Natural Resources and Forestry's Offshore Index Assessment (fall) program (Speers & Cottrill, 2008). This program involves setting standardized series of multimesh gill nets for 24 hr and collecting data on the catch. The exact dates of index netting vary within the August–October period. Three separate sites have been regularly surveyed annually from the early 1980s to the present: southern Georgian Bay (GB; mean coordinates among years: 44.6947°N, 80.6136°W; we also included in this site data from nearby Owen Sound, which was surveyed in some years), the southern Main Basin of Lake Huron (43.5605°N, 81.9143°W), and the central Main Basin of Lake Huron (44.7024°N, 81.3725°W). We pooled the two sites in the Main Basin (MB) because the distance between sites (~150 km) was small relative to the presumed scale of movement in these fishes. We assume the MB population is reproductively isolated from southern Georgian Bay, but the population structure and movement of lake whitefish among basins are not completely understood, and some degree of mixing cannot be ruled out (Eberts et al., 2017). The MB site is contained with quota management area (QMA) OH‐4 and OH‐5; the GB site is contained within QMA GB‐4 (see map in Gobin et al., 2015). Lake whitefish commercial fisheries are managed primarily through quota management, but also through gear restrictions (e.g., Caroffino & Lenart, 2017).

Previous studies have characterized growth and maturation traits using these or subsets of these data, but were not in the form we needed. Wang et al. (2008) analyzed variation in maturation schedules among multiple sites throughout the Laurentian Great Lakes, but did not analyze trends in prematuration growth rates or show the relationship between growth and maturation (i.e., the plastic effects of growth rate on maturation timing). Gobin et al. (2015) showed trends in juvenile growth rate in MB, but not in GB, and also did not directly relate cohort‐specific growth to maturation traits.

### 2.2 Growth and maturation traits

Prereproductive growth rates and maturation traits were characterized separately for each site and cohort. This cohort‐based approach generally follows that of Morgan and Colbourne (1999). All data manipulation and data analyses were performed in SAS/STAT software version 9.3 (SAS Institute Inc. 2011). To estimate prereproductive growth rate, we fit linear models to length‐at‐age data for ages 1–3. Cohort‐specific slopes and 95% confidence limits were retained if slopes were significant (≠0) at α = 0.01. For females only, logistic regression was used to estimate the age and length at 50% maturity (*A*_50_ and *L*_50_) using procedure PROBIT in SAS. Females were used to be consistent with our optimization model, and because they typically mature at an older age and larger size than males (Beauchamp et al., 2004; Wang et al., 2008). Cohort‐specific estimates of *A*_50_ and *L*_50_ were retained if the intercepts and slopes were significant (≠0) at α = 0.01. Fiducial confidence limits were calculated for *A*_50_ and *L*_50_ using procedure PROBIT in SAS. Annual variation in prereproductive growth rate, *A*_50_, and *L*_50_ were visualized using loess curves in package ggplot in R (R Core Team 2016).

We used general linear models to quantify the relationship between prematuration growth rate and maturation traits (age and length at 50% maturity). For each maturation trait, the model included cohort‐specific growth rate, the factor site, and the site × growth rate interaction. The interaction was dropped before assessing main effects (α = 0.05). A general linear model was justified on the basis of a causal relationship between maturation traits (*y*) and prematuration growth rate (*x*) (Smith, 2009). Given error in *x*, however, we also estimated the slope correction factor as var(*x*)/(var(*x*)‐var(*u*)), where var(*u*) is the estimate of variance in *x* attributed to technique error and sampling variance (Smith, 2009). In our case, var(*x*) was the variance in prereproductive growth rate (i.e., the *x* values). We used the median of the variance in the estimate of slope from the length versus age relationships to represent var(*u*).

### 2.3 Catch per unit effort

The presence of large fish has the potential to buffer populations from the undesirable evolutionary effects of fishing (Hixon, Johnson, & Sogard, 2014; Law, 2007). Thus, we extracted information on the abundance of large fish in each year. For this analysis, we used catch counts rather than fish size data, because not all fish were measured (a maximum of 50 fish are measured per deployment). Our index of abundance was catch per unit effort (CPUE) in the two largest mesh sizes (114 and 127 mm). One unit of effort was one 24 hr (approximately) multimesh gill net set, which always included one 50‐m panel each of 114‐ and 127‐mm gill net. These mesh sizes have maximum selectivities for 513 and 573 mm lake whitefish, respectively, and rarely catch fish <400 mm (Zhao & Morbey, 2017). Following Gobin et al. (2015), we calculated the annual geometric mean of ln(CPUE + 1) and the associated 95% confidence limits. This also involved adjusting catch rates of multifilament nets (used in years ≤ 1994) by 1.8 (Collins, 1979). Annual variation in CPUE was visualized using loess curves in package ggplot in R.

### 2.4 Probabilistic maturation reaction norms

For each site (GB and MB), we characterized the probabilistic maturation reaction norm (pMRN) to assess site differences. A univariate MRN describes how age or size at maturity varies with an environmental factor (e.g., growth rate) within a single genotype (Falconer & Mackay, 1996). A bivariate MRN, which is especially popular for fish, considers the plasticity of both size and age at maturity in relation to growth, and is typically shown as a line on a bivariate plot of size versus age. Variation in growth rates can cause variation in age and size at maturation along the MRN but does not cause the estimated MRN itself to change (Stearns, 1992). An extension of the MRN concept involves accounting for the inherent between‐individual variability in the timing of maturation by describing it as probabilistic. A pMRN describes the probability that an immature individual of a given age and size will mature in the following growing season (Heino, Dieckmann, & Godø, 2002). The pMRN shows the age‐specific maturation thresholds (i.e., the maturation rules) and can also be shown on a bivariate plot of size versus age.

We estimated pMRNs (*L*_p50,a_ values) for females following the procedures described in full by Barot, Heino, O'Brien, and Dieckmann (2004). Sites were analyzed separated using all the data available. In step (1), we calculated the age‐ and length‐specific probabilities of maturity conditional on being alive (age‐specific ogives, *o*[*a*,* L*_F_]). Following Ficker, Mazzucco, Gassner, Wanzenböck, and Dieckmann (2014), we first compared different logistic regression models of the maturity ogives *o*(*a*,* L*_F_) to choose among different combinations of the predictor variables age as a continuous variable (*a*), age as a factor (*a*_F_), and fork length (*L*_F_). We considered five models: (1) *a *+ ε; (2) *L*_F_ + ε; (3) *a *+ *L*_F_ + ε; (4) *a *+ *L*_F_ + *a *×* L*_F_ + ε; (5) *a*_*F*_ + *L*_F_ + *a*_F_ ×* L*_F_ + ε. To obtain reliable estimates from logistic regression, we only considered ages where the number of mature fish was ≥10, the number of immature fish was ≥10, and the total number of fish was ≥100. We chose the model with the best performance (lowest Bayesian information criterion with all variance inflation factors <10).

In step (2), we used estimates of age‐specific annual growth rate (Δ*L*_F_[*a*]) and the ogives from step (1) to estimate the age‐ and size‐specific probabilities of maturing (*m*[*a*,* L*_F_]), conditional on being alive and immature:

(1) $$m\left(a,L_{F}\right)=\frac{o\left(a,L_{F}\right)-o\left(a-1,L_{F}-\Delta L_{F}\left(a\right)\right)}{1-o(a-1,L_{F}-\Delta L_{F}\left(a\right))}$$

Age‐specific growth rates were estimated from von Bertalanffy growth functions using nonlinear regression in procedure NLIN in SAS. Values of *m*(*a*,* L*_F_) were calculated for all ages and all 1‐mm increments of *L*_F_. These values comprised the raw MRNs.

For each age *a*, a logistic regression model of *m*(*a*,* L*_F_) was used to estimate the size at which the probability of maturing equals 0.5 (*L*_p50,a_). These values of *L*_p50,a_ comprised the pMRN. Values of *m*(*a*,* L*_F_) were included in the age‐specific logistic regressions if the following conditions were met: 0 ≤ *m*(*a*,* L*_F_) ≤ 0.5 and *m*(*a*,* L*_F_) − *m*(*a*,* L*_F_‐1) > 0. This ensured that the relationship between logit(*m*) and *L*_F_ was linear (based on visual examination, at *L*_F_ values associated with *m *>* *0.5, logit[*m*] began to decelerate). To estimate confidence intervals around *L*_p50,a_, bootstrapped resampling of the observed data was performed with 1,000 replicates. Our approach to estimate pMRNs differed slightly from Wang et al. (2008) in that we estimated age‐specific growth rates from fitted von Bertalanffy models, and, as described above, applied constraints on samples sizes and values of *m*(*a*,* L*_F_) to give more reliable estimates.

To estimate pMRNs, all years were pooled because there were insufficient data for a cohort‐based approach. Thus, annual variation or trends in growth were not taken into account in Equation (1). This introduces uncertainty in the comparison of pMRNs between sites. As a result, we also tested for site differences in the maturity ogives, *o*(*a*,* L*_F_), while allowing for random intercepts among years. This was done using logistic regression in the procedure GLIMMIX in SAS, which permits the specification of random effects in logistic regression. The model included age, length, site as a fixed factor, and year as a random factor.

### 2.5 Dynamic optimization model: overview

We used a phenotypic approach to model the growth and maturation of lake whitefish as a state‐dependent energy allocation problem using a dynamic optimization model. In effect, a phenotypic approach to optimization let us determine what energy allocation strategies might be beneficial under different selection regimes, without any consideration of genetic inheritance mechanisms (for a comparison of different modeling approaches, see Kokko, 2007). Dynamic optimization models have been used widely to study behavioral or physiological decisions that depend on current state and time (e.g., time of day, day of year, or year of life) given their consequences on cumulative survival and/or reproduction at a future time (McNamara, Houston, & Collins, 2001). The model we developed closely parallels Jørgensen and Fiksen's (2006) model of energy allocation in Atlantic cod. In this model, a female allocates incoming energy to growth, storage, and reproduction in such a way to maximize the number of eggs produced over a lifetime in a stochastic environment. Females also experience different forms of mortality. Variation in lifetime patterns of growth and maturation emerge as the outcome of state‐dependent energy allocation decisions. This model has been used to explore the phenomenon of skipped spawning (Jørgensen, Ernande, Fiksen, & Dieckmann, 2006) and the fitness consequences of size‐selective fisheries for a variety of life history traits (Jørgensen et al., 2009). This type of model was well‐suited to explore the interaction between size‐selective fisheries and environmental variation in growth potential. We note that this approach does not explicitly consider ecological feedbacks that could influence somatic growth during the process of adaptation, such as negative density dependence via intraspecific competition for food.

The dynamic optimization model contains a bioenergetics submodel. This describes an individual's metabolism as well as its capacity to consume, store, and grow as functions of its current size and energy stores. These functions are fully explained in Jørgensen and Fiksen (2006). For lake whitefish, the context was a simple lake environment: Water temperature and prey availability were specified for each month of the year to reflect observed seasonal patterns.

The dynamic optimization model determines how excess energy should be used. If a fish's energy balance is positive after finding some amount of food, then the extra energy may be allocated to structural growth in length or to energy stores for future use in respiration or spawning. Every spawning month (e.g., assumed to be November for lake whitefish), the fish can convert all or none of its stored energy to eggs. Every month the fish is subject to some probability of surviving until the next month, which is the added effects of natural mortality (assumed to be invariant with respect to size and age) and size‐selective fishing mortality. Because of mortality, a trade‐off emerges between growth and storage. Allocating energy to stores in preparation for spawning is safer, in that some offspring will be produced early in life. The riskier strategy of taking time to grow larger before maturing is more rewarding in the end, but only if the fish can survive to reproduce.

An individual's fitness is the total number of offspring it produces over its lifetime. The dynamic programming algorithm finds the optimal allocation of net energy intake to storage (*u*) versus growth that maximizes the total number of eggs produced. The algorithm starts at the hypothetical last month (*t *= *T*) of the fish's life when the optimal choice for allocation (*u*) can only be “store as much as possible for the last chance to spawn.” Going backwards in time, 1 month at a time, expected fitness (*V*) is calculated for all the combinations of states the modeled fish could be in: for every starting fork length (*L*_F_ = 10–100 cm, in 2 cm increments), every starting energy stores (*E* = 0%–100% of storage capacity, in 10% increments), every food intake possible that month (ϕ = 20%–100%, in 10% increments), every allocation decision (*u* = 0%–100% of extra intake energy converted to stores, with the remainder going to structural growth, in 10% increments), and in November every spawning decision (yes/no):

(2) $$\begin{array}{cc} V(t,\;L_{F},E,\phi )= & \text{max}\left\{b\left(E\right)+S\sum_{\phi (t+1)}p\left[\phi \left(t+1\right)|\phi \left(t\right)\right]\times \right.\\ & \left.V[t+1,L_{F}\left(t+1|u\right),E\left(t+1|u\right),\phi \left(t+1\right)]\right\} \end{array}$$

*V*(*t*,* L*_F_, *E*,ϕ) is a function of fecundity *b*, survival probability *S* to the next age, the conditional probability of finding food *P* and food intake ϕ in the next time step, and *V* in the next time step. The *L*_F_(*t *+* *1) and *E*(*t *+* *1) functions are determined by the bioenergetics equations. Reproductive output via the spawning of eggs (*b*) can only occur in November. *V* is assigned as the maximum value over all possible values of *u*. The allocation decision (*u*) resulting in the highest expected fitness is stored in an optimal decision matrix. The optimal spawning decision is also stored.

Individuals are then allowed to grow and interact with the optimal decision matrices, assuming a variable environment. Environmental stochasticity is modeled as variability in food intake around an expected proportion $\overset{¯}{\chi }$ of maximum consumption *C*_max_. Thus, the forward simulation (FS) produces a random series of autocorrelated values for food intake and individual “fish” respond to these circumstances. Forward simulation reveals the optimal growth and maturation schedule.

### 2.6 Dynamic optimization model: parameterization

The dynamic optimization model was applied to Lake Huron lake whitefish and was programmed in C++ using QT Creator 3.1.1 (https://www.qt.io/ide/). This involved some modification to elements of the Atlantic cod model. First, in the bioenergetics submodel, we used the respiration and maximum consumption functions for bloater (*Coregonus hoyi*) from Rudstam, Bindowski, and Miller (1994) and applied to lake whitefish by Madenjian et al. (2006). Respiration (*R*) is a power function of body weight and also depends on temperature. In this function, we initially used a coefficient of 0.00138 as recommended by Madenjian, Pothoven, and Kao (2013). Month‐specific values of temperature (*T*, °C) were obtained from a study of lake whitefish in Lake Michigan (Madenjian et al., 2006). These values were 1.5, 0.5, 0.5, 2.0, 4.5, 6.0, 7.5, 8.0, 11.0, 9.5, 6.5, and 4.0°C, respectively, for January to December. Respiration was converted into J g^−1^ month^−1^ by multiplying *R* by 13,556 J g O_2_^−1^ and 30.5 day/month.

We used the temperature‐dependent consumption function (*C*_max_) for *Coregonus hoyi* from Binkowski and Rudstam (1994). *C*_max_ is a power function of body weight and also depends on temperature according to the function *F*(*T*) from Kitchell, Stewart, and Weininger (1977). Maximum consumption was converted into energy available for allocation by accounting for the multiplicative effects of egestion (25% of *C*_max_), excretion (10%), and the energetic cost of processing assimilated energy (specific dynamic action [SDA] = 17%). To convert to J g^−1^ month^−1^, available energy was multiplied by 30.5 day/month and month‐specific values of prey quality (J/g prey) from Madenjian et al. (2006). These values were 2,716 J/g for December to March, 2,449 J/g for April to June, 2,838 J/g for July and August, and 2,984 J/g for September to November.

Second, we assumed size selectivity fishing mortality from trap nets and gill nets, the two major types of fishing gear used to catch lake whitefish in Lake Huron. Based on data from the Ontario Ministry of Natural Resources and Forestry's commercial catch sampling program (Gile & Milne, 2006), we looked at the use of these two gear types in our sites. We selected the set of samples (i.e., deployments) targeting lake whitefish during the period 1980 to 2015. In QMA OH4‐5, gill nets and trap nets comprised 88% and 22% of the samples, respectively (*n *=* *2,951). In QMA GB4, gill nets comprised 100% of the samples (*n *=* *986). Thus, gill nets are the dominant gear type used in MB commercial fisheries, and the only gear type in GB commercial fisheries.

Size selectivity functions for gill nets and trap nets were recently estimated for Lake Huron lake whitefish (Zhao & Morbey, 2017). Size‐selective mortality from gill nets follows a double logistic function and depends on mesh size (*m* in cm):

(3) $$\begin{array}{c} S_{m}\left(L_{F}\right)=\frac{1}{1+\text{exp}\left(-\alpha_{1}\left(\frac{L_{F}}{m}-\beta_{1}\right)\right)}\times \left(1-\frac{1}{1+\text{exp}\left(-\alpha_{2}\left(\frac{L_{F}}{m}-\beta_{2}\right)\right)}\right)\times C \end{array}$$

where *S*_m_(*L*_F_) is selectivity as a function of *L*_F_, α_1_ = 6.23, β_1_ = 4.10, α_2_ = 2.87, and β_2_ = 5.09. C is a constant (=1.2398) such that maximum *S*_m_ = 1. We initially set *m* to 11.4 cm, which currently dominates in the commercial gill net fisheries in Lake Huron (maximum selectivity = 51.3 cm). Size‐selective mortality from trap nets follows a logistic function:

(4) $$\begin{array}{c} S_{t}\left(L_{F}\right)=\frac{1}{1+\text{exp}\left(-\alpha \left(L_{F}-\beta \right)\right)} \end{array}$$

where *S*_t_(*L*_F_) is trap net selectivity as a function of *L*_F_, α = 0.32 and β = 47.1 cm. This is an asymptotic function, where selectivity reaches 0.99 at 61.5 cm. Trap nets can vary in their construction, but sizes and dimensions of trap net components were not systematically recorded in the commercial catch sampling program.

A few additional modifications were made. We assumed no additional energy costs associated with movement from summer feeding areas to spawning areas, and instead assumed an additional mortality cost associated with spawning (*M*_S_). We also applied a consumption multiplier (*p*) to permit a reduction in *C*_max_ for spawners in November. We also assumed that excess energy would be stored in muscle only, rather than in muscle and liver as in Atlantic cod. Typically, muscle or whole body energy density is used to represent condition in lake whitefish (Muir et al., 2010; Pothoven et al., 2001, 2006), and lipid content (% by dry mass) in age 5+ lake whitefish is generally lower in liver (10%–15%) than in all somatic tissue (15%–30%; Johnston et al., 2012).

Additional parameters were required for functions relating stored energy to weight (ε, *K*_max_, *K*_min_, *L*_STD_, ρ_*E*_), the conversion of net energy intake to growth and stores (δ_growth_, δ_store_, and ρ_*S*_), and the conversion of stored energy to eggs (κ_5_;Appendix 1). For some of these, we were able to estimate or approximate values using data for Lake Huron lake whitefish. When data were not available, we kept the original values used by Jørgensen and Fiksen (2006). We assumed a value of 0.2 year^−1^ for instantaneous natural mortality rate (*M*), which led to a large range of ages in our modeled population in the absence of fisheries and spawning mortality. Under constant age‐specific mortality, *M *=* *0.2 corresponds to a 50% reduction in cohort size by age 3.5 years and a 95% reduction in cohort size by age 15 years.

**Figure 1:**
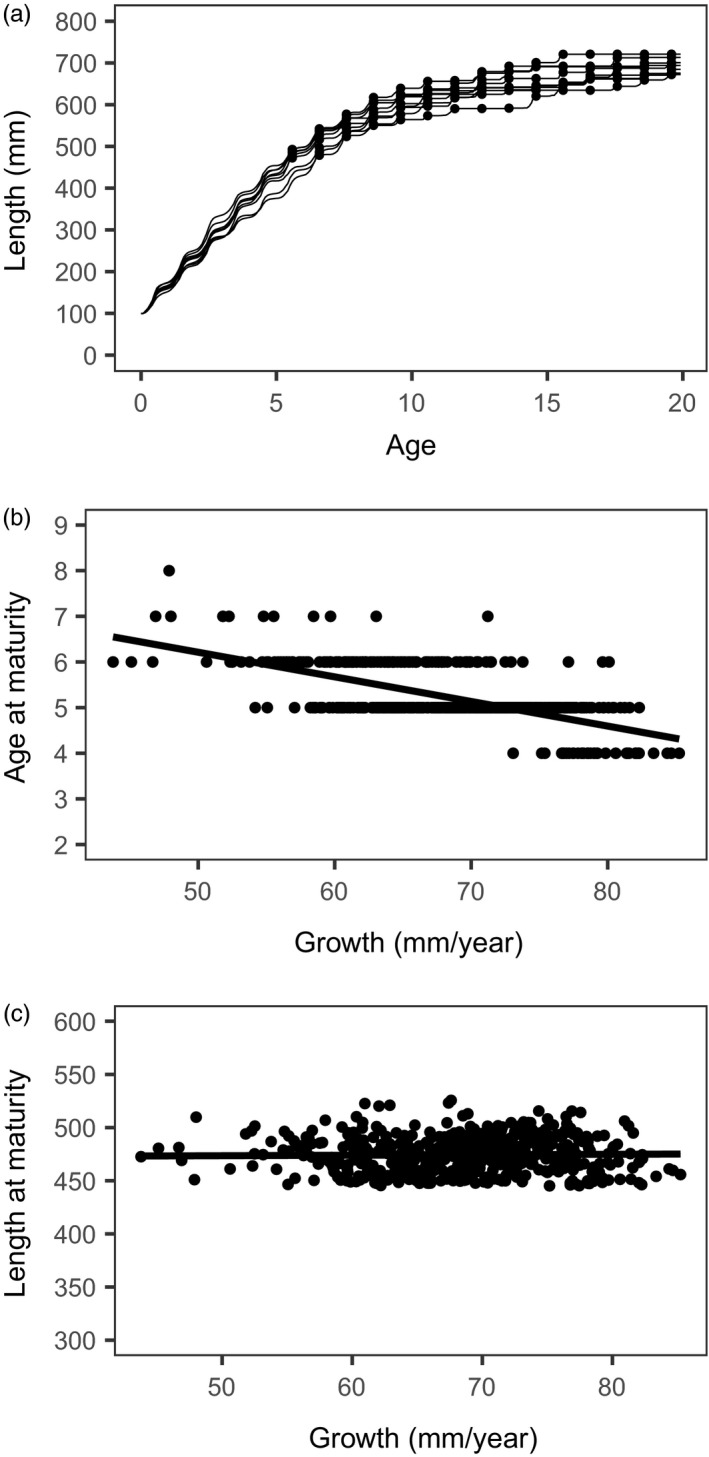
Output of the state‐dependent energy allocation model for 500 simulated lake whitefish using baseline parameters. (a) Growth trajectories for the first 10 simulated fish, showing the seasonal pattern of length increment and the months of November when the optimal decision was to spawn (solid symbols). (b) The relationship between age at maturation and estimated age 1–3 growth rate (mm/year) for all simulated fish. (c) The relationship between length at maturation (mm) and estimated age 1–3 growth rate (mm/year) for all simulated fish. The linear regression lines are shown in panels (b) and (c)

After generating the decision matrices for optimal allocation *u* and spawning, forward simulation was used to extract information about size at maturity. In each FS, we let individuals (*n *=* *50) grow and interact with the decision matrices. All environmental and bioenergetics parameters were the same as those used in the backward iteration phase. The output of a FS included age (converted into years), *L*_F_ (converted to mm), and spawning decision (to spawn or not). Simulated individuals followed different growth trajectories owing to environmental stochasticity, but matured at similar lengths regardless of their experienced growth rate. The transition from being immature to mature was discrete with spawning occurring nearly every year thereafter (Figure 1). In effect, the optimal maturation reaction norm was flat and could be characterized by the mean length at maturation (*L*_m_). For each simulation, we calculated mean *L*_m_ for the 50 individuals (the standard deviation of *L*_m_ was small, ranging from about 1–3 mm).

### 2.7 Model calibration

Given inevitable uncertainty in the parameters values used in the DP, we adjusted some parameter values so that maturation occurred at realistic sizes for lake whitefish. Prior to calibration, the DP systematically underestimated size at maturity relative to observed *L*_50_ values. To increase the benefit of delayed maturation, we first increased the costs of spawning (*M*_s_ and *p*). These were parameters for which we had no a priori information, and costs of migration and spawning are important components of the Atlantic cod model (Jørgensen & Fiksen, 2006). We also adjusted the coefficient of the respiration function, which seemed to have a large effect on the model output.

### 2.8 Model sensitivity

We assessed the sensitivity of optimal *L*_m_ to fish growth rate, fishing mortality rate, and gear type. To induce differences in growth, we varied the energetic cost of processing food (SDA) from 0.085 (50% of 0.17) to 0.34 (200% of 0.17) by increments of 0.0425. Based on simulated growth trajectories, increasing SDA simulated a reduction in age 1–3 growth rate from 76 mm/year (when SDA = 0.085) to 55 mm/year (when SDA = 0.34). Other parameters affecting energy intake (e.g., $\overset{¯}{\chi }$) are expected to affect maturation schedules in a similar way as SDA. Instantaneous fishing mortality (*F*) for maximally selected fish was arbitrarily set to 0, 0.1, 0.2, or 0.3 year^−1^ to show a range of responses. Gear type was set to be gill net or trap net. For gill nets, size selectivity was a function of mesh size, and we set mesh size to 89 mm (3.5 inches; maximum selectivity at 403 mm) or 114 mm (4.5 inches; maximum selectivity at 513 mm; Zhao & Morbey, 2017). Trap nets of different variants have not been described or modeled for lake whitefish fisheries.

## 3 RESULTS

### 3.1 Growth, maturation traits, and CPUE

**Figure 2:**
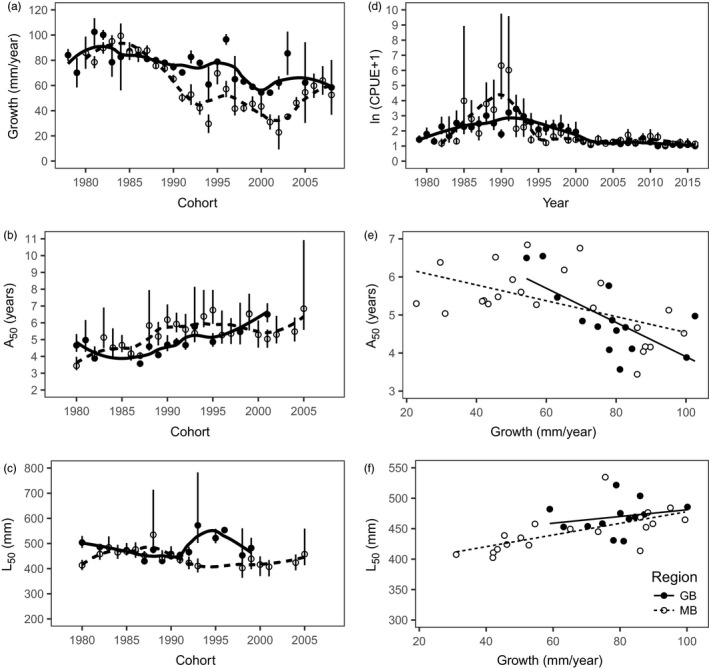
Patterns in the growth, maturation traits, and catch per unit effort of lake whitefish in Georgian Bay (GB; solid symbols) and the Main Basin of Lake Huron (MB; open symbols). On the left, cohort‐specific values are shown for (a) age 1‐3 growth rate, (b) age at 50% maturity, and (c) length at 50% maturity. In (d), catch per unit effort in 114‐mm and 127‐mm gill nets is shown for each year. Also shown in (e) are the relationship between age at 50% maturity and growth rate and (f) the relationship between length at 50% maturity and growth rate. Loess curves are shown in panels (a), (b), (c), and (d), whereas the linear regression lines are shown in panels (e) and (f). The 95% confidence limits are shown in panels (a) and (d); the 95% fiducial limits are shown in panels (b) and (c)

Lake whitefish showed some differences in growth rate between the two Lake Huron sites. In both sites, prematuration growth rate was variable among cohorts and showed a declining trend across the time series (Figure 2a). In MB, the 1983 and 1984 cohorts had the highest growth rates; in GB, the 1981 and 1982 cohorts had the highest growth rates. Growth rate decline appeared to be more severe in MB than in GB, with evidence of improving growth rates in MB among cohorts after 2002. In MB, the CPUE of lake whitefish in large mesh gill nets (114 and 127 mm) was highest in 1990 and 1991 (Figure 2d). In GB, peak CPUE was lower than in MB and occurred slightly later in 1991 and 1992. In both sites, CPUE was consistently low from the year 2000 onwards.

Age at 50% maturity (*A*_50_) was variable among cohorts and showed an increasing trend in both sites (Figure 2b). Length at 50% maturity (*L*_50_) was also variable among cohorts, but did not display any obvious long‐term trend in either site (Figure 2c). Variability in *A*_50_ and *L*_50_ was consistent with life history plasticity, whereby faster growing fish mature at younger ages (Figure 2e) and larger sizes (Figure 2f). In the general linear model of *A*_50_, the effect of growth rate was significant (slope [β] = −0.025, *t*_35_ = −3.99, *p *=* *.0003) but site was not (*F*_1,35_ = 0.03, *p *>* *.5; overall *r* ^2^ = .35), and there was no significant site × growth rate interaction (*F*_1,34_ = 2.21, *p *=* *.15). The slope correction factor was small (GB: 1.010; MB: 1.014). In the general linear model of *L*_50_, the effect of growth rate was significant (β = 0.989, *t*_31_ = 3.11, *p *=* *.004). *L*_50_ did not differ between sites (*F*_1,31_ = 3.55, *p *=* *.069; overall *r* ^2^ = .40), but there was a trend toward higher *L*_50_ in GB than in MB. There was no significant site × growth rate interaction (*F*_1,30_ = 0.05, *p *>* *.5). The slope correction factor was small (GB: 1.017; MB: 1.012).

### 3.2 Probabilistic maturation reaction norms

**Figure 3:**
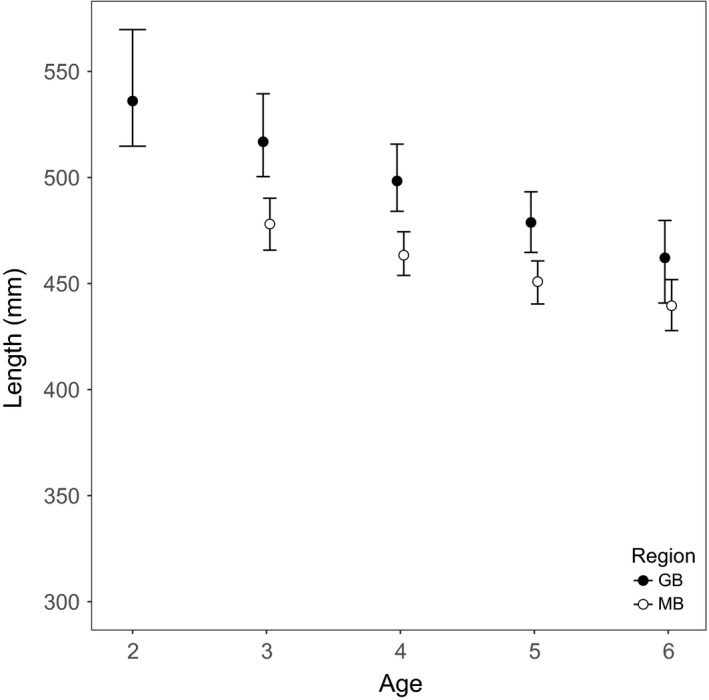
Probabilistic maturation reaction norms for female lake whitefish from Georgian Bay (GB; solid symbols) and the Main Basin of Lake Huron (MB; open symbols). At each age *a*,* L*_p50,a_ is shown along with the 95% confidence limits from bootstrap resampling. MB lake whitefish had smaller threshold sizes for maturing than those from GB, and in both sites, the pMRN was negatively sloped

The estimated pMRNs suggest site differences in maturation thresholds. In the estimation of pMRNs, the best‐performing statistical models of the maturity ogives *o*(*a*,* L*_F_) used age and length as continuous variables, meaning that fish were more likely to be mature if they were older and larger for their age. *L*_p50,a_ values could be estimated for ages 2–6 in GB and ages 3–6 in MB, and showed that pMRNs were higher in GB than in MB (Figure 3). The slopes of the pMRNs were slightly negative in both sites (Figure 3).

When testing for site differences in the maturity ogives, there were overall effects of age (β = 0.616, *t*_20,592_ = 13.0, *p *<* *.0001), length (β = 0.022, *t*_20,592_ = 26.5, *p *<* *.0001), and site (β_GB vs. MB_ = −0.784, *t*_71_ = −3.4, *p *=* *.001; model intercept = −12.196). Thus, for any age and length combination, GB females had a lower probability of being mature than MB females. This is consistent with larger size thresholds for maturation in GB than in MB. For example, at age 4, the model estimates a length at 50% maturity of 470.4 mm in GB and 435.4 mm in MB.

### 3.3 Dynamic optimization model

Calibration of the DP was required so that optimal *L*_m_ better approximated observed *L*_50_ values. When we included additional costs associated with spawning (*M*_S_ = 0.3 and *p *=* *.25) and a lower value for the respiration coefficient (0.001 instead of 0.00138), optimal *L*_m_ was 476 ± 1.6 mm when *F *=* *0 and SDA = 0.17. With additional costs associated with spawning, lake whitefish delay spawning and instead allocate resources to growth to achieve a higher body size. A lower respiration coefficient helped to increase growth rate. Other explanations for the precalibration mismatch between model and data, such as a minimum required size for sexual maturation, likely would produce a similar effect to that of higher *M*_S_ and *p*.

**Figure 4:**
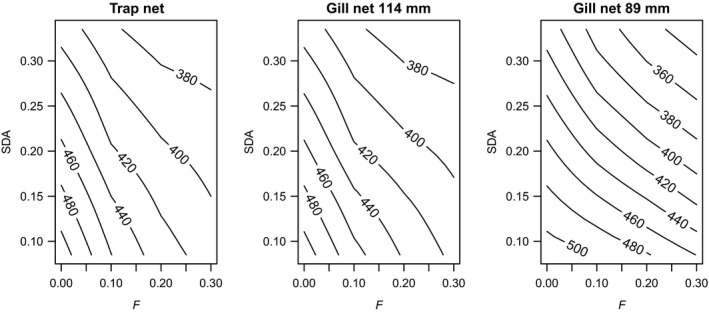
The predicted, long‐term evolutionary effects of mortality and growth regimes based on a state‐dependent, dynamic optimization model of energy allocation for Lake Huron lake whitefish for trap nets (a), 114‐mm gill nets (b), and (c) 89‐mm gill nets. Fishing mortality rate (*F*) is shown on the *x*‐axis, and the energetic cost of assimilating food (specific dynamic action or SDA) is shown on the *y*‐axis. The contours show predicted lengths at maturity (mm), which were estimated from locally weighted smoothing

Fishing mortality and fish growth rate both affected optimal *L*_m_. When controlling for gear type and SDA, increasing fishing mortality from 0 to 0.3 year^−1^ invariably reduced optimal *L*_m_ (Figure 4). When controlling for gear type and *F*, lowering growth rate by increasing SDA invariably reduced optimal *L*_m_ (Figure 4). Higher SDA favors lake whitefish that mature at a lower size threshold because slower growth lowers the fecundity advantage associated with delaying maturity.

The magnitude of the effect of increasing fishing mortality depended on gear type and fish growth rate. For trap nets and 114 mm gill nets, increasing fishing mortality reduced optimal *L*_m_ across all values of SDA*,* but to a greater degree at lower values of SDA (fast growth; Figure 4a,b). Optimal *L*_m_ was always lower than maximum selectivity for these two gear types (513 mm for 114 mm gill nets; >615 mm for trap nets). When fishing with 89 mm gill nets, fishing mortality more strongly reduced optimal *L*_m_ at high values of SDA (slow growth) and had less effect on optimal *L*_m_ at low values of SDA (fast growth; Figure 4c). Buffering occurred at low values of SDA because fish could quickly outgrow the sizes when they were most vulnerable to gill nets and mature at a larger size. Optimal *L*_m_ was higher than maximum selectivity (403 mm) for a wide range of low *F* and low SDA values.

## 4 DISCUSSION

We used a dynamic optimization model to explore the long‐term evolutionary effects of different growth and mortality regimes on maturation size thresholds in lake whitefish. When we simulated a low growth selection regime by increasing the energetic cost of assimilating food (specific dynamic action or SDA), the model predicted a smaller optimal size threshold for maturation. When we simulated size‐selective fishing using realistic functions for gill nets and trap nets, an increase in fishing mortality also favored a smaller optimal size threshold for maturation. These general predictions were not unexpected and do not differ from those generated by deterministic life history models (e.g., Roff, 1984). Moreover, growth and mortality regimes account for the local adaptation of maturation traits among populations of lake whitefish (Beauchamp et al., 2004), and we expect both to contribute to contemporary adaptation of maturation schedules.

In the model, the long‐term evolutionary effects of fishing with different gear types depended on the growth regime. When fishing with trap nets, the optimal response to higher fishing mortality was to mature at a smaller size, and this effect was magnified under a fast growth regime. With trap nets, smaller fish are less vulnerable to fishing owing to the sigmoid‐shaped selectivity of this gear type (Zhao & Morbey, 2017). Fish that mature at smaller sizes have lower reproductive output, but they also avoid growing into vulnerable size classes where their survival would be low. In contrast to trap nets, gill nets present two size refuges for fish, and the presence of an upper size refuge has the potential to buffer a population from the long‐term evolutionary effects of fishing (Jørgensen et al., 2009; Law, 2007). However, this was not an outcome for Lake Huron lake whitefish when fishing with 114‐mm gill nets, the mesh size currently dominating in the commercial fishery. Buffering was a possible outcome with the smaller mesh gill net (89 mm), but only when growth potential was high. Under these conditions, fish could quickly outgrow the period of vulnerability to harvest and then mature at a large size with high fecundity. When growth potential is low, however, fishing with the 89‐mm gill net favors maturity at smaller sizes than would occur with a larger mesh size.

The growth rates experienced in the two sites have implications for the evolutionary outcome of size‐selective fisheries. Within both Lake Huron sites, growth rates were variable among cohorts and generally declined across the time series. Trends of declining growth have been reported at multiple sites in the Laurentian Great Lakes and have been attributed to changing food webs (Ebener, 2013; Fera, Rennie, & Dunlop, 2015; Gobin et al., 2015; Lumb, Johnson, Cook, & Hoyle, 2007; Rennie et al., 2009). Through plasticity, a reduction in growth was associated with an increase in age at 50% maturity and a decrease in length at 50% maturity. The estimated pMRNs had shallow negative slopes in both sites. Overall, most of the estimated size thresholds for maturation (and the cohort‐specific *L*_50_ values in Figure 2c) were intermediate between the sizes of maximally selected fish for 89‐mm (403 mm) and 114‐mm gill nets (513 mm). According to the dynamic optimization model, 89‐mm gill nets have the potential to buffer fisheries‐induced selection on *L*_m_ by mostly targeting small immature fish, but only in a fast growth environment. In a slow growth environment, the use of 89‐mm gill nets could have the opposite effect by intensifying selection for a reduced size threshold for maturation. Fishing with 114‐mm gill nets targets larger, mature fish and should select for a reduced size threshold for maturation, regardless of growth rate.

We expect the two sites in Lake Huron to differ in their evolutionary sensitivity to size‐selective fisheries. Overall, GB fish grew faster and matured at a younger age and larger size than MB fish. Whereas the slope of the pMRN was similar between sites, its elevation was somewhat higher in GB fish. A higher elevation pMRN in GB could be the result of local adaptation to higher growth rates in GB, although other selection factors could be important. According to the dynamic optimization model, the evolutionary effects of increasing *F* should differ depend on growth rate and gear type. In a population already adapted to low growth (e.g., MB), fisheries‐induced selection as a consequence of fishing with 114 mm gill nets is expected to be weaker (the contour lines are farther apart with increasing *F*) than in a population already adapted to high growth (e.g., GB). This pattern reverses, however, if fishing with 89‐mm gill nets. This suggests that a strategy of using smaller mesh gill nets to protect large, fecund fish might be more effective in GB than in MB.

Dynamic optimization models determine optimal phenotypes at evolutionary equilibrium given a defined selection environment and do not make specific predictions about short‐term evolutionary rates. The predicted outcomes for maturation size thresholds would only be realized if selection differentials were consistently high over multiple generations, and if there was sufficient heritability of state‐based energy allocation and spawning decisions. In Lake Huron, lake whitefish populations experience highly variable selection environments both in terms of growth rate (e.g., Gobin et al., 2015) and fishing mortality (e.g., Ebener et al., 2008). In the commercial lake whitefish fisheries in northern Main Basin of Lake Huron, for example, instantaneous fishing mortality from trap nets and gill nets ranged from about 0.05–0.69 (mean = 0.31) during 1976 to 2015 (page 42, Caroffino & Lenart, 2017). Sea lamprey predation can be another major source of size‐selective mortality for contemporary populations (Caroffino & Lenart, 2017), although the specific form of size‐selective mortality imposed by sea lampreys is not straightforward given that mortality depends on the probability of attack (positively related to fish size) and the probability of survival given an attack (negatively related to fish size; Bence et al., 2003). In addition, lake whitefish can be targeted by recreational fishing. Thus, evolutionary trajectories of maturation thresholds might proceed at highly variable rates in nature.

Certain factors have the potential to buffer or counteract evolutionary change in maturation schedules. For example, fisheries‐induced evolution can be buffered by negative density dependence in somatic growth rate that is the outcome of intraspecific competition for limiting resources (Dunlop et al., 2015; Gobin et al., 2016). On the one hand, Healey (1980) provides experimental evidence of fisheries‐induced, negative density‐dependent growth at the whole lake level in small lakes, and Lorenzen and Enberg (2002) discuss more generally the importance of considering negative density‐dependent growth as a population regulation mechanism in fish populations. On the other hand, Gobin et al. (2016) show an overall positive relationship between lake whitefish biomass and juvenile growth in QMA OH4‐5. They further suggest that site‐specific changes in lake whitefish density may be driven more so by variation in habitat quality, which could determine growth rate and carrying capacity, than to variation in fishing mortality. Positive density‐dependent processes may also play a role. For example, in a spatially and temporally heterogeneous environment, habitat selection by mobile predators could lead to positive correlations between habitat quality, somatic growth rate, and density. Further research about density‐dependent behavior and movement would help to reduce uncertainty about density effects at the different spatial scales relevant to lake whitefish.

Selection also may target intrinsic growth rate or one of the bioenergetic determinants of prematuration growth rate (e.g., maximum consumption, *C*_max_). For example, long‐term fishing pressure lead to the evolution of slower growth rates in *Coregonus palaea* in a small lake, where fishing is regulated by a minimum size limit (Nusslé et al., 2009). In experimental studies of captive *Menidia menidia*, harvesting of large fish leads to the evolution of slower growth rates, whereas harvesting of small fish leads to the evolution of faster growth rates (Conover & Munch, 2002). Evolutionary models show that fishing mortality can favor higher or lower growth rate, depending on the size selectivity of fishing mortality and other factors (Dunlop, Heino et al., 2009; Eikeset et al., 2016).

Concern about fisheries‐induced evolution has pervaded discourse on fisheries management and poses important questions about whether (and how) fisheries‐induced evolution should be controlled or reversed (e.g., Dunlop et al., 2015; Heino et al., 2013). A frequent recommendation is to protect large fecund fish, which could be done, for example, by fishing using a gear type with bell‐shaped size selectivity (Hixon et al., 2014; Law, 2007). Small mesh gill nets in particular can favor delayed maturation, extend the age and size distribution of the population, and result in good fisheries yield in the long term (Jørgensen et al., 2009; Zimmerman & Jørgensen, 2017). However, fishing with small mesh gill nets may intensify evolution toward smaller size thresholds for maturation under poor growth or high mortality regimes. Whether or not this happens will depend on the particular fishery and the biology of the target species. In unpredictable and uncertain selection regimes, reliance on small mesh gill nets to favor delayed maturation might be a risky strategy (Jørgensen et al., 2009). A more cautious option for protecting large, fecund fish would be to keep overall mortality rates low, either through further controls of fishing or sea lamprey predation. For example, Hutchings (2009) advocates for fishing below a threshold level of fishing mortality (*F*_evol_) that would favor delayed over early maturity. These values of *F*_evol_ can be calculated for different stocks with different growth rates, mortality regimes, and gear types.

Our model provides insight about the potential for gill nets to buffer lake whitefish populations from the long‐term evolutionary effects of fishing. Compared to trap nets, gill nets are expected to provide an upper size refuge for fish. Fish that grow through vulnerable stages to mature at a larger size will be more fecund over many years and could potentially help to buffer populations from low recruitment. However, this is not a general rule. When considering the specific case of Lake Huron whitefish, we predicted very similar evolutionary effects of fishing with trap nets and 114‐mm gill nets. Buffering only occurred with the smaller mesh gill net and only in a high growth, low mortality environment.

## CONFLICT OF INTEREST

None declared.

## APPENDIX 1

### Parameters used for the dynamic optimization model of maturation in lake whitefish

**Table:**

Parameter	Value and unit	Explanation and source
ε	0.211	Component of the allometric scaling coefficient in *W* ∝ *L* ^3+ε^, where *W *= body mass (g) and *L *= fork length (cm)( )Estimated from Lake Huron lake whitefish data
*K*_min_	0.75	Minimum condition factor at *L*_std_ From Jørgensen and Fiksen (2006)
*K*_max_	1.25	Maximum condition factor at *L*_std_ From Jørgensen and Fiksen (2006)
*L*_std_	32 cm	Length for which *K*_min_ and *K*_max_ are defined Average *L*_F_ from Lake Huron lake whitefish data
ρ_*E*_	5,520 J/g	Energy density of muscle energy stores Lake whitefish estimate from Muir et al. (2014)
ρ_*S*_	4,000 J/g	Energy density of all somatic tissue From Jørgensen and Fiksen (2006)
χ	0.75	Expected proportion of maximum food intake From Jørgensen and Fiksen (2006)
*C*_1_	0.9	Autocorrelation coefficient in food intake function From Jørgensen and Fiksen (2006)
*C*_2_	0.15	Variance scaling factor in food intake function From Jørgensen and Fiksen (2006)
Δ*L*_max_	20 cm/year	Maximum growth rate of *L*_F_ Approximated from Lake Huron lake whitefish data
δ_store_	0.4	Assimilation efficiency for the conversion of ingested energy to stores From Jørgensen and Fiksen (2006)
δ_growth_	0.08	Assimilation efficiency for growth of somatic structures From Jørgensen and Fiksen (2006)
κ_5_	0.256 eggs/J	Conversion between stored energy and spawned eggs From Jørgensen and Fiksen (2006)
*M*	0.2 year^−1^	Instantaneous natural mortality Assumed
*M*_*S*_	0.3 year^−1^	Additional instantaneous mortality during the month of spawning Calibrated
*p*	0.25	Consumption multiplier during month of spawning Calibrated
*T*	384 months	Maximum age in the model
*L*_min_	10 cm	Length of fish at *t *=* *0 in forward simulation (April)
